# Impact of COVID‐19 pandemic on epilepsy care in Japan: A national‐level multicenter retrospective cohort study

**DOI:** 10.1002/epi4.12616

**Published:** 2022-06-12

**Authors:** Naoto Kuroda, Takafumi Kubota, Toru Horinouchi, Naoki Ikegaya, Yu Kitazawa, Satoshi Kodama, Izumi Kuramochi, Teppei Matsubara, Naoto Nagino, Shuichiro Neshige, Temma Soga, Yutaro Takayama, Daichi Sone, IMPACT‐J EPILEPSY (In‐depth Multicenter analysis during Pandemic of Covid‐19 Throughout Japan for EPILEPSY practice) study group, Kousuke Kanemoto, Akio Ikeda, Kiyohito Terada, Hiroko Goji, Shinji Ohara, Koichi Hagiwara, Takashi Kamada, Koji Iida, Nobutsune Ishikawa, Hideaki Shiraishi, Osato Iwata, Hidenori Sugano, Yasushi Iimura, Takuichiro Higashi, Hiroshi Hosoyama, Ryosuke Hanaya, Akihiro Shimotake, Takayuki Kikuchi, Takeshi Yoshida, Hiroshi Shigeto, Jun Yokoyama, Takahiko Mukaino, Masaaki Kato, Masanori Sekimoto, Masahiro Mizobuchi, Yoko Aburakawa, Masaki Iwasaki, Eiji Nakagawa, Tomohiro Iwata, Kentaro Tokumoto, Takuji Nishida, Yukitoshi Takahashi, Kenjiro Kikuchi, Ryuki Matsuura, Shin‐ichiro Hamano, Ayataka Fujimoto, Hideo Enoki, Kyoichi Tomoto, Masako Watanabe, Youji Takubo, Toshihiko Fukuchi, Hidetoshi Nakamoto, Yuichi Kubota, Naoto Kunii, Yuichiro Shirota, Eiichi Ishikawa, Nobukazu Nakasato, Taketoshi Maehara, Motoki Inaji, Shunsuke Takagi, Takashi Enokizono, Yosuke Masuda, Takahiro Hayashi

**Affiliations:** ^1^ Japan Young Epilepsy Section (YES‐Japan) Tokyo Japan; ^2^ Department of Pediatrics, Wayne State University Detroit Michigan USA; ^3^ Department of Neurology, University Hospitals of Cleveland Medical Center Case Western Reserve University Cleveland Ohio USA; ^4^ Department of Psychiatry and Neurology Hokkaido University Graduate School of Medicine Sapporo Japan; ^5^ Department of Neurosurgery, Graduate School of Medicine Yokohama City University Yokohama Japan; ^6^ Department of Neurology and Stroke Medicine Yokohama City University Graduate School of Medicine Yokohama Japan; ^7^ Department of Neurology, Graduate School of Medicine The University of Tokyo Tokyo Japan; ^8^ Department of Psychiatry, Saitama Medical Center Saitama Medical University Saitama Japan; ^9^ Athinoula A. Martinos Center for Biomedical Imaging Massachusetts General Hospital Charlestown Massachusetts USA; ^10^ Epilepsy Center, TMG Asaka Medical Center Saitama Japan; ^11^ Department of Clinical Neuroscience and Therapeutics, Hiroshima University Graduate School of Biomedical and Health Sciences Hiroshima Japan; ^12^ Department of Epileptology Tohoku University Graduate School of Medicine Miyagi Japan; ^13^ Department of Neurosurgery, National Center Hospital National Center of Neurology and Psychiatry Tokyo Japan; ^14^ Department of Clinical and Experimental Epilepsy UCL Institute of Neurology London UK

**Keywords:** epilepsy center, hospitalization, neurology, neurosurgery, SARS‐CoV‐2

## Abstract

**Objective:**

The impact of the coronavirus disease 2019 (COVID‐19) pandemic on epilepsy care across Japan was investigated by conducting a multicenter retrospective cohort study.

**Methods:**

This study included monthly data on the frequency of (1) visits by outpatients with epilepsy, (2) outpatient electroencephalography (EEG) studies, (3) telemedicine for epilepsy, (4) admissions for epilepsy, (5) EEG monitoring, and (6) epilepsy surgery in epilepsy centers and clinics across Japan between January 2019 and December 2020. We defined the primary outcome as epilepsy center‐specific monthly data divided by the 12‐month average in 2019 for each facility. We determined whether the COVID‐19 pandemic‐related factors (such as year [2019 or 2020], COVID‐19 cases in each prefecture in the previous month, and a state of emergency) were independently associated with these outcomes.

**Results:**

In 2020, the frequency of outpatient EEG studies (−10.7%, *P* < .001) and cases with telemedicine (+2608%, *P* = .031) were affected. The number of COVID‐19 cases was an independent associated factor for epilepsy admission (−3.75 × 10^−3^% per case, *P* < .001) and EEG monitoring (−3.81 × 10^−3^% per case, *P* = .004). Furthermore, a state of emergency was an independent factor associated with outpatient with epilepsy (−11.9%, *P* < .001), outpatient EEG (−32.3%, *P* < .001), telemedicine for epilepsy (+12,915%, *P* < .001), epilepsy admissions (−35.3%; *P* < .001), EEG monitoring (−24.7%: *P* < .001), and epilepsy surgery (−50.3%, *P* < .001).

**Significance:**

We demonstrated the significant impact that the COVID‐19 pandemic had on epilepsy care. These results support those of previous studies and clarify the effect size of each pandemic‐related factor on epilepsy care.

**Key points**

- We investigated the impact of the coronavirus disease 2019 (COVID‐19) pandemic on epilepsy care by conducting a national‐level retrospective cohort study.
- A state of emergency due to the COVID‐19 pandemic, as well as the number of COVID‐19 cases in each prefecture, had tremendous effects on epilepsy care.
- Therefore, these results may have implications for future policies during pandemics.
- In addition, this method allows us to predict the impact of COVID‐19 or other pandemics on epilepsy care, for the future.

## 1 INTRODUCTION

The coronavirus disease 2019 (COVID‐19) pandemic has afflicted multiple medical fields, including epilepsy care. The relationship between epilepsy care and COVID‐19 has been explored previously.^1^, ^2^ For instance, the promotion and spread of telemedicine in the field of epilepsy care from 2020 are attributed to the COVID‐19 pandemic.^2^, ^3^, ^4^, ^5^, ^6^ Additionally, postponement of elective surgery was recommended during the early phase of the pandemic.^7^, ^8^ Limited access to healthcare is a risk factor for the worsening of seizures,^9^ and at the beginning of the COVID‐19 pandemic, some societies recommended that electroencephalography (EEG) examinations should be reduced to a minimum to limit patient–staff contact.^10^, ^11^To reiterate, the COVID‐19 pandemic has considerably impacted several aspects of epilepsy care, as reported by researchers across countries.^5^, ^12^, ^13^, ^14^, ^15^, ^16^, ^17^, ^18^, ^19^, ^20^

However, almost all such studies are based on descriptive or pre–post quasi‐experimental study designs, which solely compared the numbers related to epilepsy care before and during the COVID‐19 pandemic. Due to such a study design, one cannot determine the effect size of the number of COVID‐19 cases or a state of emergency independently from the effect size throughout 2020. We hypothesized that each pandemic‐related factor would independently impact epilepsy care. Based on this hypothesis, we determined the effect of the COVID‐19 pandemic‐related factors on epilepsy care by conducting a nationwide multicenter retrospective cohort study, initiated by the Japan Young Epilepsy Section (YES‐Japan), which is a national chapter of the Young Epilepsy Section of the International League Against Epilepsy (ILAE‐YES). Data acquisition was conducted by the study group, In‐depth Multicenter analysis during Pandemic of Covid‐19 Throughout Japan for EPILEPSY care (IMPACT‐J EPILEPSY). Specifically, we investigated whether, and to what extent, epilepsy care was limited in 2020 (with the first case of COVID‐19 in Japan being reported in January 2020). We also investigated whether and to what extent the number of COVID‐19 patients or policies, such as a state of emergency, independently limited epilepsy care. It is key to note that the total frequency of COVID‐19 cases in Japan as of December 31st, 2020, was 235 907 (the transition is shown in Figure S1). A detailed understanding of the impact of the pandemic on epilepsy care, including the magnitude of the impact of COVID‐19 itself, plus a policy of state of emergency, would provide important clues for the continued provision of epilepsy care during the ongoing pandemic. Such information would also impact the level of preparedness in future pandemics.

## 2 MATERIALS AND METHODS

### 2.1 Study design and data collection

The present nationwide, multicenter, retrospective cohort study complied with the guidelines for reporting observational studies.^21^ This study adhered to the principles of the Declaration of Helsinki. The study was registered at each participating hospital/clinic in line with applicable regulations. The study was approved by the ethics committee (#A201200010) of Yokohama City University, which was the initiating facility, along with other facilities requiring approval from a suitable ethics committee (Methods S1). We sent a call for participating facilities through the official email list of the Japanese Epilepsy Society. A total of 4 facilities specializing in epilepsy care and 20 hospitals accredited as epilepsy centers or epilepsy training facilities across all Japanese regions agreed to participate (Figure S2). These participating facilities cover 9/17 (53%) of epilepsy centers across Japan. Informed consent was not obtained from individual patients because this study did not collect individual patient data. Data were obtained by collaborators in each participating facility using either medical records or health insurance bills, and reported to YES‐Japan.

### 2.2 Primary outcomes definition and data collection

We collected six measurements that reflect the activity of epilepsy care at each facility in Japan as follows: the number of (1) visits by outpatients with epilepsy (including both in‐person and telemedicine visits; counting every visit if a patient visited multiple times in the same month); (2) outpatient EEG studies (including both epilepsy‐related and nonepilepsy‐related tests but not including ambulatory EEG because ambulatory EEG is not approved in Japan); (3) cases of telemedicine for epilepsy (including both phone calls and video visits); (4) admissions for epilepsy (including elective admission for EEG monitoring, drug adjustment, or introduction of dietary therapy, and emergency admissions); (5) long‐term EEG monitoring (including EEG monitoring at least over 24 hours with or without video recording, and in‐or‐out of epilepsy monitoring units)^22^, ^23^; and (6) epilepsy surgery. We collected monthly data on the number of outpatients with epilepsy visits, outpatient EEG studies, and telemedicine cases of epilepsy in a total of 24 facilities between January 2019 and December 2020. We collected monthly data on the number of admissions for epilepsy, EEG monitoring, and epilepsy surgery between January 2019 and December 2020 from 20, 19, and 17 hospitals, respectively, because these procedures were not available in all clinics and hospitals.

We defined the primary outcome as epilepsy center‐specific monthly data divided by the 12‐month average in 2019 for each facility. Based on the collected data, we normalized each monthly measurement by dividing it by the 12‐month average in 2019 in each facility. Regarding the number of outpatient visits with telemedicine, we used only the data of 6/24 facilities, as the 12‐month average of the number of outpatient visits with telemedicine in 2019 was 0 in the other 18/24 facilities. Regarding epilepsy surgery, we used only data from hospitals with six or more epilepsy surgeries in 2019 (15/17 hospitals).

### 2.3 Definition of variables related to activity of epilepsy care

We defined three variables related to the activity of epilepsy care that were unrelated to the impact of the COVID‐19 pandemic, as follows: (a) medical facility status (hospital or clinic), (b) population of each prefecture in which each facility is located, and (c) number of physicians with epilepsy board certificates. The given variables were chosen based on the following assumptions: (a) the role of facility is different between hospitals and clinics, (b) the prevalence proportion of patients with epilepsy is widely known to be 0.7%–1.0%; hence, prefectures with higher populations have a greater number of patients with epilepsy; and finally, (c) facilities with many physicians with epilepsy board certificates can provide more epilepsy‐related medical care.

### 2.4 Definition of variables that reflect the impact of the COVID‐19 pandemic

We defined the impact of COVID‐19 on society using three variables. One variable is (d) 2019 or 2020, to consider the impact of the COVID‐19 pandemic throughout the whole year of 2020. The other, (e) the total number of COVID‐19 cases in each region in the previous month. This variable was collected from the open‐access information (https://web.sapmed.ac.jp/canmol/coronavirus/japan.html).^24^ We incorporated this variable based on the hypothesis that a greater number of COVID‐19 cases in a previous month on each region confers pressure on usual medical practice in a month on the region. The last variable is (f) the presence of a state of emergency in Japan because this restricts people/patients' activities, including access to healthcare providers. We incorporated a state of emergency (April–May 2020 in Japan) as a variable, based on the hypothesis that the impact of a state of emergency was independent of the number of COVID‐19 positive cases.^25^, ^26^, ^27^, ^28^ Specifically, we dichotomized the presence of a state of emergency (April 2020 to May 2020) and the absence of a state of emergency (other periods). A state of emergency is a situation in which a government is empowered to be able to implement governmental policies that it would normally not be permitted (such as stay‐at‐home orders), for the safety and protection of its citizens/nations. A state of emergency is declared in cases of war, terrorism, pandemics, or other emergency issues. During the COVID‐19 pandemic, many countries/state governments declared a state of emergency.

### 2.5 Statistical analysis

We performed a linear mixed‐model analysis using SPSS v27 (IBM) to determine the factors associated with each of the six epilepsy‐related data measurements. For outcomes of the number of visits by outpatients with epilepsy, outpatient EEG studies, and cases of telemedicine in epilepsy, the fixed‐effect factors unrelated to the pandemic included: (a) the type of facility (hospital/clinic), (b) the population in each prefecture, and (c) physicians with epilepsy board certificates in each facility. Fixed‐effect factors related to the COVID‐19 pandemic included: (d) whether they were present in 2020 or 2019, (e) COVID‐19 cases in each prefecture in the previous month, and (f) whether they were present during a state of emergency (April–May 2020, in Japan), to consider the independent effect of each variable. For outcomes of the number of admissions for epilepsy, EEG monitoring, and epilepsy surgery, the fixed‐effect factors did not include (a) the type of facility (hospital/clinic) because these practices were not performed in clinics. The random‐effect factors included the intercept addressing variability among facilities.

### 2.6 Subgroup analysis

To identify subgroups that were more affected by the COVID‐19 pandemic in epilepsy care, we performed subgroup analyses of the frequency of visits by outpatients with epilepsy and cases of telemedicine in epilepsy.

The outcome of the number of visits by outpatients with epilepsy was divided into two subgroups: the number of *new visits* by outpatients with epilepsy and the number of *follow‐up visits* by outpatients with epilepsy. Similarly, we divided the number of cases of epilepsy treated with telemedicine into two groups: the number of *new visits* and the number of *follow‐up visits*.

We performed a linear mixed‐model analysis for these subgroups, in a manner similar to the primary outcomes. Regarding telemedicine, we used only the data of 2/24 facilities for the analysis of *new visits* and 4/24 facilities for the analysis of *follow‐up visits*. This is because the 12‐month average of the number of outpatient visits with telemedicine in 2019 was 0 in 22/24 facilities for *new visits* and 20/24 facilities for *follow‐up visits*.

## 3 RESULTS

### 3.1 Descriptive analysis and monthly transition for each outcome

The mean and standard deviation of the monthly number across the 24 facilities in each year were as follows: (1) epilepsy outpatient: 14 266 ± 631 (2019) and 13 965 ± 1019 (2020); (2) EEG outpatient: 2541 ± 308 (2019) and 2117 ± 409 (2020); (3) telemedicine: 200 ± 20 (2019) and 1769 ± 1018 (2020); (4) epilepsy admission: 642 ± 60 (2019) and 554 ± 86 (2020); (5) EEG monitoring: 476 ± 40 (2019) and 460 ± 48 (2020); and (6) epilepsy surgery: 58 ± 10 (2019) and 54 ± 9 (2020).

**FIGURE 1:**
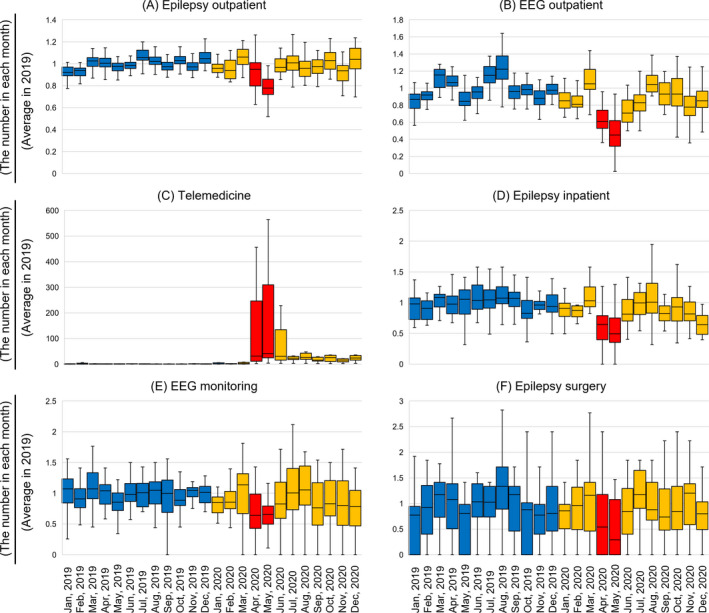
Box plots of monthly data values for each primary outcome. The blue boxplots show the data for 2019. The orange boxplots show the data for 2020. The red boxplots show the data during a state of emergency (April–May 2020). (A) Normalized data of the monthly number of outpatient visits with epilepsy by the 12‐month average in 2019 in each facility. (B) Normalized data of the monthly number of outpatient electroencephalography (EEG) studies by 12‐month average in 2019 in each facility. (C) Normalized data of the monthly number of cases of telemedicine in epilepsy by 12‐month average in 2019 in each facility. (D) Normalized data of the monthly number of epilepsy admissions by 12‐month average in 2019 in each facility. (E) Normalized data of the monthly number of EEG monitoring by 12‐month average in 2019 in each facility. (F) Normalized data of the monthly number of epilepsy surgery by 12‐month average in 2019 in each facility

Figure 1 shows the monthly transition for the primary outcomes (epilepsy center‐specific monthly data divided by the 12‐month average in 2019 for each facility) for each of the six epilepsy‐related data measurements. In 2019, telemedicine was found to be negligible. Additionally, an increase or decrease is visible in each trend during a state of emergency.

### 3.2 Identification of independent associated factors with each outcome

**FIGURE 2:**
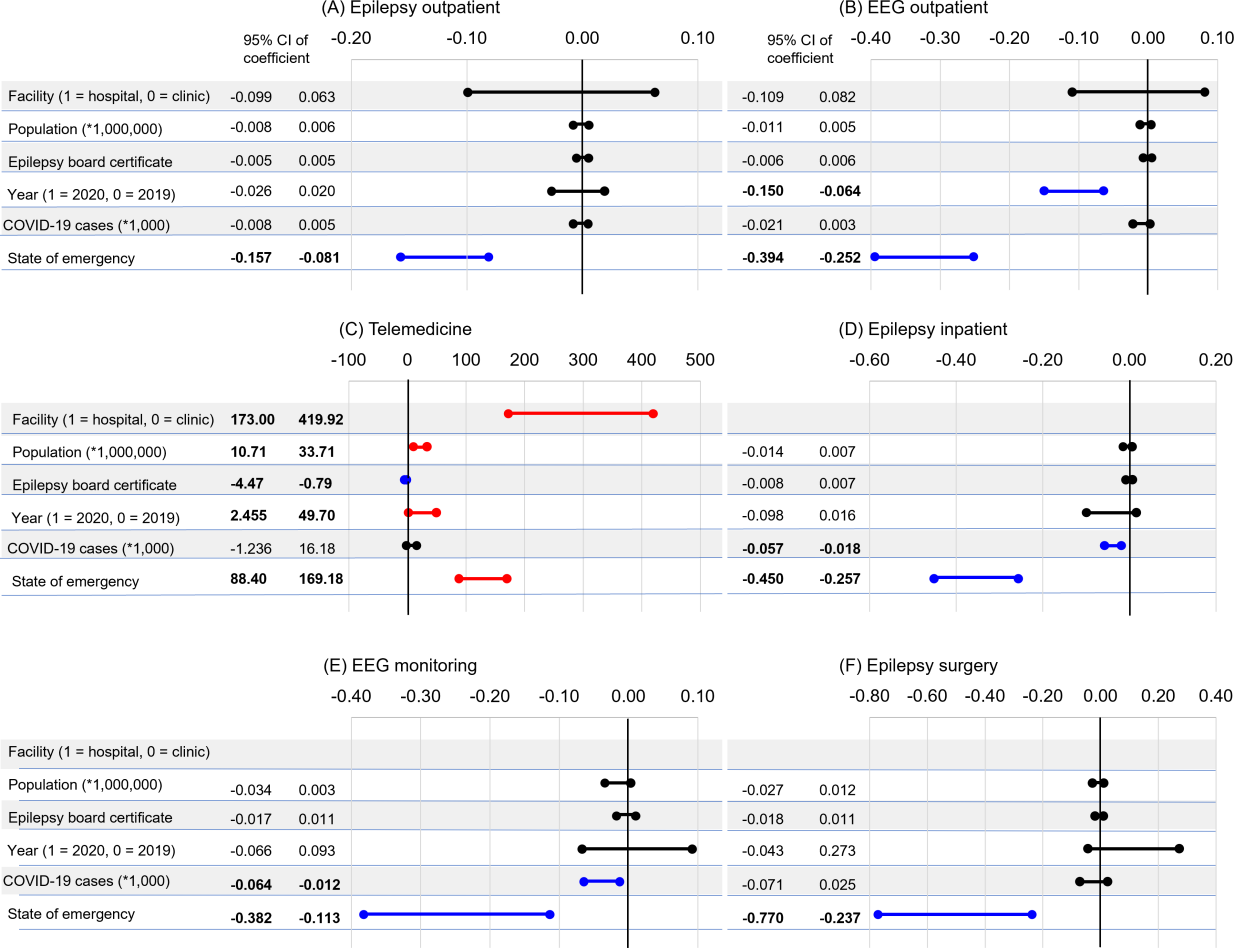
Results of linear mixed‐model analysis for each outcome and graphical summary of the 95% confidence interval of the coefficients of the independent variables. Variables with red plots and bars, whose 95% confidence interval of the estimate of coefficient calculated with the linear mixed‐model analysis is over 0, representing the positively associated factors with each outcome. Variables with blue plots and bars, whose 95% confidence interval of the estimate of the coefficient is under 0, represent the factors negatively associated with each outcome. (A) Visits by outpatients with epilepsy. (B) Outpatient electroencephalography (EEG) studies. (C) Telemedicine cases with epilepsy patients. (D) Admissions for epilepsy. (E) EEG monitors. (F) Epilepsy surgeries. The figures show the coefficients multiplied by 1 000 000 for the effect size of the population number in each prefecture and by 1000 for the effect size of the number of COVID‐19 cases in each prefecture in the previous month, respectively. CI, confidence interval

The number of EEG outpatients was significantly reduced, and telemedicine increased tremendously in 2020 (EEG outpatients: effect size = −10.7%, *P* < .001; telemedicine: effect size = +2608%, *P* = .031) (Figure 2, Table S1). The number of COVID‐19 cases in the previous month was a significantly independent associated factor for admission and EEG monitoring (admissions: effect size = −3.75 × 10^−3^% per case, *P* < .001; EEG monitoring: effect size = −3.81 × 10^−3^% per case, *P* = .004). A state of emergency was an independent factor that significantly affected all six aspects of epilepsy care (outpatient visits: effect size = −11.9%, *P* < .001; EEG outpatient: effect size = −32.3%, *P* < .001; telemedicine: effect size = +12 915%, *P* < .001; admissions: effect size = −35.3%, *P* < 0.001; EEG monitoring: effect size = −24.7%, *P* < 0.001; epilepsy surgery: effect size = −50.3%, *P* < 0.001). Figure 2 summarizes the 95% confidence intervals of the linear mixed‐model effect sizes of each variable for each outcome. This shows that a state of emergency is an associated factor independent of the Year 2020 and COVID‐19 cases. The effect size of a state of emergency was greater than that of other factors in all aspects of epilepsy care except telemedicine.

### 3.3 Subgroup analysis

Figure S3 shows the monthly transitions for the subgroup analysis. In the subgroup of outpatients with epilepsy, both the number of *new visits* and *follow‐up visits* were significantly reduced by the COVID‐19 pandemic (Table S2). In the telemedicine subgroup, the number of *follow‐up visits* increased in 2020 and during a state of emergency significantly, although the number of *new visits* was not (Table S3).

## 4 DISCUSSION

### 4.1 Summary and interpretation of our findings

We investigated whether the Year 2020, the number of COVID‐19 cases, and a state of emergency had an impact on epilepsy care in Japan using monthly data from 24 facilities for 24 consecutive months. Our models demonstrated that these COVID‐19‐related variables independently affected epilepsy care. The results allow us to understand the factors of the COVID‐19 pandemic and the extent to which it impacted epilepsy care. Therefore, our results have implications for future policies during pandemics.

Our results demonstrated that the COVID‐19 pandemic reduced both the number of epilepsy outpatient visits and admissions, similar to the results reported in studies across the world.^14^, ^19^, ^20^ Both the number of outpatient visits (−11.9%) and admissions (−35.3%) in epilepsy centers were decreased during a state of emergency. The number of COVID‐19 cases in each prefecture was also an independent suppressive predictor for epilepsy admissions (COVID‐19 cases: −3.75 × 10^−3^% [in other words, an increase of 1000 positive cases had an impact of −3.75%]). Subgroup analysis for outpatient visits showed that both the number of *new* and *follow‐up patients* were reduced by the COVID‐19 pandemic. In the case of patients with stable seizures, some societies have recommended that medication be supplied to reduce the chance of patients coming to the healthcare facility physically during the early phase of the pandemic.^29^ Therefore, the decrease in the number of follow‐up patients could be the result of each healthcare facility's compliance with the recommended medication prescription. However, the decrease in the number of new outpatients may be due to other medical institutions refraining from or postponing referring patients to epilepsy centers or clinics due to the COVID‐19 pandemic. This includes the possibility that due to the COVID‐19 pandemic, patients in need of appropriate diagnosis and treatment did not receive the necessary and timely medical care. In addition, it would be necessary to promote telemedicine in both new and follow‐up patients to allow all outpatients to be examined, even during the pandemic.

As shown in our results, the number of telemedicine cases increased in the year 2020 and during a state of emergency. The promotion of telemedicine during the pandemic has been reported in many studies in the field of epilepsy care.^3^, ^14^, ^19^ Another notable result of our study was that the type of facility had a tremendous effect on the increase in telemedicine in epilepsy outpatients. This would be because only one was included from clinics in the analysis, and the clinic had 23.2 telemedicine cases in the 12‐month average in 2019, although four of the five hospitals included in the analysis had less than two cases in the 12‐month average in 2019. Due to this difference, monthly telemedicine data divided by the 12‐month average in 2019 would be estimated to be lower in clinics than in hospitals. Subgroup analysis of telemedicine cases showed that telemedicine for *follow‐up patients* was greatly promoted by the pandemic, whereas telemedicine for *new patients* was not. This was thought to be largely influenced by governmental telemedicine regulations. Prior to the pandemic, telemedicine was rarely promoted, and it was principally allowed to be used solely for follow‐up. During the COVID‐19 pandemic, the Japanese government urgently approved the use of telemedicine in both *new patients* and *follow‐up patients* in 2020 to take full advantage of contactless medicine.^30^ However, according to their statement, there are more requirements to be met with *new patients* than with *follow‐up patients*.^31^ Specifically, for *new patients*, to prevent identity theft, there are several procedures to verify their identity and insurance identification. Our results indicate that telemedicine for new patients should be promoted to continue providing proper medical resources to patients with epilepsy during this continuing pandemic.

Our results demonstrated that both outpatient EEG and EEG monitoring were affected by the pandemic. Several studies have reported that the COVID‐19 pandemic has reduced the chances of EEG (including long‐term monitoring) evaluation.^14^, ^17^, ^19^, ^20^ The interpretation of this result could be that some societies' guidance about the indication of EEG during the early phase of the pandemic would have affected the restriction of EEG worldwide.^10^, ^11^

Similar to the outpatient, admission, and EEG results, the number of epilepsy surgeries was also affected by the pandemic. The number of epilepsy surgeries decreased by 50.3%. The suppressive impact of the pandemic on epilepsy surgery has been reported similar to our results, in some studies.^17^, ^19^ The reason might be that elective surgeries, which are preferentially postponed during the COVID‐19 pandemic, comprise the majority of epilepsy surgeries. Another reason could be that many cases of epilepsy surgery require presurgical evaluations such as outpatient EEG or EEG monitoring, which were also reduced during the COVID‐19 pandemic.

This study successfully determined the suppressive impact of the number of COVID‐19 cases and a state of emergency on epilepsy care. The impact of policy implementation during a pandemic, such as a state of emergency, has not so far been investigated in the field of epilepsy care. Several studies in other medical fields have reported that such policies have affected medical care.^25^, ^26^ Within Japan, medical institutions had fewer visits, and medical functions were suppressed in other medical fields under a state of emergency.^27^, ^28^ Our study showed that a state of emergency in Japan may have had a similar impact on epilepsy care. Even though the stay‐home order was not legally binding when a state of emergency was declared, unlike lockdowns,^32^ it was observed that the declaration had a significant impact on epilepsy care.

### 4.2 Methodological considerations

Our study has several strengths. First, this is one of the large‐scale studies conducted on the impact of the COVID‐19 pandemic on epilepsy care by obtaining monthly data on epilepsy clinics from 24 collaborating facilities from January 2019 to December 2020. Second, we evaluated multiple aspects of epilepsy care using six outcomes. Third, and most importantly, we could independently estimate the effect size of the year, 2020, the number of COVID‐19 cases, and a state of emergency using linear mixed‐model analysis. Our study is novel in successfully and independently evaluating each variable, such as the year 2020, number of COVID‐19 cases, and a state of emergency. This method allows us to predict the impact of the COVID‐19 pandemic, or other pandemics in the future, on epilepsy care, applying variables including the year of the pandemic, the number of infected cases, and a state of emergency to this model.

Furthermore, the study has some limitations. The first limitation is the generalization of these results to other countries. In this study, we collected data on epilepsy care in Japan. The impact of the COVID‐19 pandemic on society has varied across countries. The detailed rules, effectiveness, and influence of a state of emergency also vary from country to country. In Japan, a state of emergency was offered in the very early phase in terms of COVID‐19 cases (the Japanese government offered an emergency state on April 16, 2020, when the number of new COVID‐19 cases across Japan was just 563). During a state of emergency, the Japanese government had no legal limitations or restrictions on nations but strongly recommended staying at home. The availability of telemedicine varies depending on a country's infrastructure. The number of epilepsy surgeries and EEG monitoring depends on the level of medical care provided in the country. The second limitation is that our model could not cover all the variables. For example, the number of COVID‐19 patients admitted to a hospital directly restricts their epilepsy care.^17^ Another lacking covariate in this analysis was geographical variations in epilepsy care and telemedicine access during the study period. The availability and benefits of telemedicine depend on geographical variations.^33^ In addition, socioeconomic variables in each region or each patient were not incorporated in this analysis. It is known that socioeconomic variables affect healthcare inequity in epilepsy care.^34^, ^35^Another well‐known thing is that the impact of the COVID‐19 pandemic is greater in people with lower socioeconomic backgrounds.^36^, ^37^ Third, we did not collect the clinical outcomes of individual patients. Further studies are needed to determine whether the suppressive effect of the COVID‐19 pandemic on epilepsy care affects patient outcomes. Fourth, our study data were obtained from either medical records or health insurance bills. We were not able to make the data requirement method consistent, which might cause selection and reporting bias. Some research reported discrepancies in the retrospective medical chart and the health insurance linkage data.^38^, ^39^, ^40^ Finally, although we covered 53% of epilepsy centers in Japan, our study design evaluated data limited to 4 facilities specializing in epilepsy care and 20 hospitals accredited as epilepsy centers or epilepsy training facilities. Therefore, our results might not be reflective of the patients with undiagnosed epilepsy or community settings, such as patients with nonsevere epilepsy seen by family physicians. For further study, random sampling throughout Japan, including the community setting or clinics not specializing in epilepsy care, might be better to reflect the impact on epilepsy care in the community setting across Japan.

Our nationwide multicenter retrospective cohort study demonstrated that a state of emergency due to the COVID‐19 pandemic, as well as the number of COVID‐19 cases in each region, had tremendous effects on epilepsy care. The findings of this study should be interpreted in the context of the study design.

## CONFLICT OF INTEREST

None of the authors has any conflict of interest to disclose. We confirm that we have read the journal's position on issues involved in ethical publication and affirm that this report is consistent with the guidelines.

## Supporting information


Appendix S1

## APPENDIX 1

IMPACT‐J EPILEPSY: Kousuke Kanemoto (Neuropsychiatric Department, Aichi Medical University, Aichi, Japan). Akio Ikeda (Department of Epilepsy, Movement Disorders and Physiology, Kyoto University Graduate School of Medicine, Kyoto, Japan). Kiyohito Terada (Yokohama Minoru Epilepsy & Developmental Clinic, Kanagawa, Japan). Hiroko Goji (Neuropsychiatric Department, Aichi Medical University, Aichi, Japan). Shinji Ohara (Department of Neurosurgery, Fukuoka Sanno Hospital, Fukuoka, Japan). Koichi Hagiwara (Epilepsy and Sleep Center, Fukuoka Sanno Hospital, Fukuoka, Japan). Takashi Kamada (Epilepsy and Sleep Center, Fukuoka Sanno Hospital, Fukuoka, Japan). Koji Iida (Department of Neurosurgery, Graduate School of Biomedical and Health Sciences, Epilepsy Center, Hiroshima University, Hiroshima, Japan). Nobutsune Ishikawa (Department of Pediatrics, Graduate School of Biomedical and Health Sciences, Epilepsy Center, Hiroshima University, Hiroshima, Japan). Hideaki Shiraishi (Department of Pediatrics, Epilepsy Center, Hokkaido University Hospital, Hokkaido, Japan). Osato Iwata (Iwata Clinic, Tokyo, Japan). Hidenori Sugano (Department of Neurosurgery, Epilepsy Center, Juntendo University, Tokyo, Japan). Yasushi Iimura (Department of Neurosurgery, Epilepsy Center, Juntendo University, Tokyo, Japan). Takuichiro Higashi (Department of Neurosurgery, Graduate School of Medical and Dental Sciences, Kagoshima University, Kagoshima, Japan). Hiroshi Hosoyama (Department of Neurosurgery, Graduate School of Medical and Dental Sciences, Kagoshima University, Kagoshima, Japan). Ryosuke Hanaya (Department of Neurosurgery, Graduate School of Medical and Dental Sciences, Kagoshima University, Kagoshima, Japan). Akihiro Shimotake (Department of Neurology, Graduate School of Medicine, Kyoto University, Kyoto, Japan). Takayuki Kikuchi (Department of Neurosurgery, Graduate School of Medicine, Kyoto University, Kyoto, Japan). Takeshi Yoshida (Department of Pediatrics, Graduate School of Medicine, Kyoto University, Kyoto, Japan). Hiroshi Shigeto (Division of Medical Technology, Department of Health Sciences, Graduate School of Medical Sciences, Kyushu University, Fukuoka, Japan). Jun Yokoyama (Department of Neurology, Neurological Institute, Graduate School of Medical Sciences, Kyushu University, Fukuoka, Japan). Takahiko Mukaino (Department of Neurology, Neurological Institute, Graduate School of Medical Sciences, Kyushu University, Fukuoka, Japan). Masaaki Kato (Musashino Kokubunji Clinic, Tokyo, Japan). Masanori Sekimoto (Musashino Kokubunji Clinic, Tokyo, Japan). Masahiro Mizobuchi (Department of Neurology, Nakamura Memorial Hospital, Hokkaido, Japan). Yoko Aburakawa (Department of Neurology, Nakamura Memorial Hospital, Hokkaido, Japan). Masaki Iwasaki (Department of Neurosurgery, National Center Hospital, National Center of Neurology and Psychiatry, Tokyo, Japan). Eiji Nakagawa (Department of Child Neurology, National Center Hospital, National Center of Neurology and Psychiatry, Tokyo, Japan). Tomohiro Iwata (Department of Psychiatry, National Defense Medical College, Saitama, Japan). Kentaro Tokumoto (Epilepsy Center, NHO Shizuoka Institute of Epilepsy and Neurological Disorders, Shizuoka, Japan). Takuji Nishida (Epilepsy Center, NHO Shizuoka Institute of Epilepsy and Neurological Disorders, Shizuoka, Japan). Yukitoshi Takahashi (Epilepsy Center, NHO Shizuoka Institute of Epilepsy and Neurological Disorders, Shizuoka, Japan). Kenjiro Kikuchi (Division of Neurology, Saitama Children's Medical Center, Saitama, Japan). Ryuki Matsuura (Division of Neurology, Saitama Children's Medical Center, Saitama, Japan). Shin‐ichiro Hamano (Division of Neurology, Saitama Children's Medical Center, Saitama, Japan). Ayataka Fujimoto (Comprehensive Epilepsy Center, Seirei Hamamatsu General Hospital, Shizuoka, Japan). Hideo Enoki (Comprehensive Epilepsy Center, Seirei Hamamatsu General Hospital, Shizuoka, Japan). Kyoichi Tomoto (Department of Neurosurgery, Seirei Hamamatsu General Hospital, Shizuoka, Japan). Masako Watanabe (Shinjuku Neuro Clinic, Tokyo, Japan). Youji Takubo (Shinjuku Neuro Clinic, Tokyo, Japan). Toshihiko Fukuchi (Suzukake Clinic, Aichi, Japan). Hidetoshi Nakamoto (Epilepsy Center, TMG Asaka Medical Center, Saitama, Japan). Yuichi Kubota (Epilepsy Center, TMG Asaka Medical Center, Saitama, Japan). Naoto Kunii (Department of Neurosurgery, Graduate School of Medicine, The University of Tokyo, Tokyo, Japan). Yuichiro Shirota (Department of Clinical Laboratory Medicine, Graduate School of Medicine, The University of Tokyo, Tokyo, Japan). Eiichi Ishikawa (Department of Neurosurgery, Faculty of Medicine, University of Tsukuba, Ibaraki, Japan). Nobukazu Nakasato (Department of Epileptology, Tohoku University Graduate School of Medicine, Miyagi, Japan). Taketoshi Maehara (Department of Neurosurgery, Tokyo Medical and Dental University, Tokyo, Japan). Motoki Inaji (Department of Neurosurgery, Tokyo Medical and Dental University, Tokyo, Japan). Shunsuke Takagi (Department of Psychiatry and Behavioral Sciences, Tokyo Medical and Dental University, Tokyo, Japan). Takashi Enokizono (Department of Pediatrics, University of Tsukuba Hospital, Ibaraki, Japan). Yosuke Masuda (Department of Neurosurgery, University of Tsukuba Hospital, Ibaraki, Japan). Takahiro Hayashi (Department of Neurosurgery, Yokohama City University, Graduate School of Medicine, Kanagawa, Japan).

## References

[1] Kuroda N . Epilepsy and COVID‐19: updated evidence and narrative review. Epilepsy Behav. 2021;116:107785. 33515934 10.1016/j.yebeh.2021.107785 PMC7805398

[2] Cross JH , Kwon CS , Asadi‐Pooya AA , Balagura G , Gómez‐Iglesias P , Guekht A , et al. Epilepsy care during the COVID‐19 pandemic. Epilepsia. 2021;62:2322–32. 34428314 10.1111/epi.17045 PMC8652685

[3] Blanco EC , Centeno M , Tio E , Muriana D , Peñas JJG , Serrano P , et al. Emergency implementation of telemedicine for epilepsy in Spain: results of a survey during SARS‐CoV‐2 pandemic. Epilepsy Behav. 2020;111:107211. 32540769 10.1016/j.yebeh.2020.107211 PMC7274642

[4] Panda PK , Dawman L , Panda P , Sharawat IK . Feasibility and effectiveness of teleconsultation in children with epilepsy amidst the ongoing COVID‐19 pandemic in a resource‐limited country. Seizure. 2020;81:29–35. 32712376 10.1016/j.seizure.2020.07.013 PMC7368411

[5] von Wrede R , Moskau‐Hartmann S , Baumgartner T , Helmstaedter C , Surges R . Counseling of people with epilepsy via telemedicine: experiences at a German tertiary epilepsy center during the COVID‐19 pandemic. Epilepsy Behav. 2020;112:107298. 32801068 10.1016/j.yebeh.2020.107298 PMC7422810

[6] Willems LM , Balcik Y , Noda AH , Siebenbrodt K , Leimeister S , McCoy J , et al. SARS‐CoV‐2‐related rapid reorganization of an epilepsy outpatient clinic from personal appointments to telemedicine services: a German single‐center experience. Epilepsy Behav. 2020;112:107483. 33181898 10.1016/j.yebeh.2020.107483 PMC7537633

[7] Iacobucci G . Covid‐19: all non‐urgent elective surgery is suspended for at least three months in England. BMJ. 2020;368:m1106. 32188602 10.1136/bmj.m1106

[8] Burke JF , Chan AK , Mummaneni V , Chou D , Lobo EP , Berger MS , et al. Letter: the coronavirus disease 2019 global pandemic: a neurosurgical treatment algorithm. Neurosurgery. 2020;87(1):E50–6. 32242901 10.1093/neuros/nyaa116 PMC7184344

[9] Mostacci B , Licchetta L , Cacciavillani C , Di Vito L , Ferri L , Menghi V , et al. The impact of the COVID‐19 pandemic on people with epilepsy. An Italian Survey and a global perspective. Front Neurol. 2020;11:613719. 33391172 10.3389/fneur.2020.613719 PMC7775598

[10] San‐Juan D , Jiménez CR , Camilli CX , de la Cruz Reyes LA , Galindo EGA , Burbano GER , et al. Guidance for clinical neurophysiology examination throughout the COVID‐19 pandemic. Latin American chapter of the IFCN task force – COVID‐19. Clin Neurophysiol. 2020;131(7):1589–98. 32417701 10.1016/j.clinph.2020.04.011 PMC7252108

[11] Canadian Society of Clinical Neurophysiologists (CSCN) , Canadian Association of Electroneurophysiology Technologists (CAET) , Association of Electromyography Technologists of Canada (AETC) , Board of Registration of Electromyography Technologists of Canada (BRETC) , Canadian Board of Registration of Electroencephalograph Technologists (CBRET) , et al. Practice Guidelines for Canadian Neurophysiology Laboratories during the COVID‐19 pandemic. Can J Neurol Sci. 2021;48(1):25–30. 32811585 10.1017/cjn.2020.184 PMC7578631

[12] Huang S , Wu C , Jia Y , Li G , Zhu Z , Lu K , et al. COVID‐19 outbreak: the impact of stress on seizures in patients with epilepsy. Epilepsia. 2020;61(9):1884–93. 32761900 10.1111/epi.16635 PMC7436883

[13] Rosengard JL , Donato J , Ferastraoaru V , Zhao D , Molinero I , Boro A , et al. Seizure control, stress, and access to care during the COVID‐19 pandemic in New York City: the patient perspective. Epilepsia. 2021;62(1):41–50. 33258109 10.1111/epi.16779 PMC7753328

[14] Albert DVF , Das RR , Acharya JN , Lee JW , Pollard JR , Punia V , et al. The impact of COVID‐19 on epilepsy care: a survey of the American Epilepsy Society Membership. Epilepsy Curr. 2020;20(5):316–24. 32942901 10.1177/1535759720956994 PMC7502678

[15] Fonseca E , Quintana M , Lallana S , Luis Restrepo J , Abraira L , Santamarina E , et al. Epilepsy in time of COVID‐19: a survey‐based study. Acta Neurol Scand. 2020;142(6):545–54. 32799337 10.1111/ane.13335 PMC7460986

[16] Koh MY , Lim KS , Fong SL , Khor SB , Tan CT . Impact of COVID‐19 on quality of life in people with epilepsy, and a multinational comparison of clinical and psychological impacts. Epilepsy Behav. 2021;117:107849. 33631434 10.1016/j.yebeh.2021.107849 PMC8021335

[17] Rathore C , Baheti N , Bansal AR , Jabeen SA , Gopinath S , Jagtap S , et al. Impact of COVID‐19 pandemic on epilepsy practice in India: a tripartite survey. Seizure. 2021;86:60–7. 33550135 10.1016/j.seizure.2020.12.025 PMC7837209

[18] Sanchez‐Larsen A , Gonzalez‐Villar E , Díaz‐Maroto I , Layos‐Romero A , Martínez‐Martín Á , Alcahut‐Rodriguez C , et al. Influence of the COVID‐19 outbreak in people with epilepsy: analysis of a Spanish population (EPICOVID registry). Epilepsy Behav. 2020;112:107396. 32911299 10.1016/j.yebeh.2020.107396 PMC7476448

[19] Wirrell EC , Grinspan ZM , Knupp KG , Jiang Y , Hammeed B , Mytinger JR , et al. Care delivery for children with epilepsy during the COVID‐19 pandemic: an international survey of clinicians. J Child Neurol. 2020;35:924–33. 32666891 10.1177/0883073820940189 PMC7364331

[20] Assenza G , Lanzone J , Ricci L , Boscarino M , Tombini M , Galimberti CA , et al. Electroencephalography at the time of Covid‐19 pandemic in Italy. Neurol Sci. 2020;41(8):1999–2004. 32588368 10.1007/s10072-020-04546-8 PMC7316521

[21] von Elm E , Altman DG , Egger M , Pocock SJ , Gøtzsche PC , Vandenbroucke JP , et al. The Strengthening the Reporting of Observational Studies in Epidemiology (STROBE) statement: guidelines for reporting observational studies. Lancet. 2007;370(9596):1453–7. 18064739 10.1016/S0140-6736(07)61602-X

[22] Logar C , Walzl B , Lechner H . Role of long‐term EEG monitoring in diagnosis and treatment of epilepsy. Eur Neurol. 1994;34(Suppl 1):29–32. 10.1159/000119506 8001606

[23] Cascino GD , Trenerry MR , So EL , Sharbrough FW , Shin C , Lagerlund TD , et al. Routine EEG and temporal lobe epilepsy: relation to long‐term EEG monitoring, quantitative MRI, and operative outcome. Epilepsia. 1996 Jul;37(7):651–6. 8681897 10.1111/j.1528-1157.1996.tb00629.x

[24] Idogawa M , Tange S , Nakase H , Tokino T . Interactive web‐based graphs of coronavirus disease 2019 cases and deaths per population by country. Clin Infect Dis. 2020;71(15):902–3. 32339228 10.1093/cid/ciaa500 PMC7197615

[25] Westgard BC , Morgan MW , Vazquez‐Benitez G , Erickson LO , Zwank MD . An analysis of changes in emergency department visits after a state declaration during the time of COVID‐19. Ann Emerg Med. 2020;76(5):595–601. 33008651 10.1016/j.annemergmed.2020.06.019 PMC7287464

[26] Mitchell RD , O'Reilly GM , Mitra B , Smit V , Miller JP , Cameron PA . Impact of COVID‐19 state of emergency restrictions on presentations to two Victorian emergency departments. Emerg Med Australas. 2020;32(6):1027–33. 32748481 10.1111/1742-6723.13606 PMC7436380

[27] Toyoda Y , Katanoda K , Ishii K , Yamamoto H , Tabuchi T . Negative impact of the COVID‐19 state of emergency on breast cancer screening participation in Japan. Breast Cancer. 2021;28:1340–5. 34241799 10.1007/s12282-021-01272-7 PMC8267509

[28] Komatsu H , Banno K , Yanaihara N , Kimura T , Board Members of Japan Society of Obstetrics and Gynecology . Prevention and practice during the COVID‐19 emergency declaration period in Japanese obstetrical/gynecological facilities. J Obstet Gynaecol Res. 2020;46(11):2237–41. 32909322 10.1111/jog.14432

[29] Kuroda N . Epilepsy and COVID‐19: associations and important considerations. Epilepsy Behav. 2020;108:107122. 32361677 10.1016/j.yebeh.2020.107122 PMC7174174

[30] Kubota T , Kuroda N , Horinouchi T , Ikegaya N , Kitazawa Y , Kodama S , et al. Barriers to telemedicine among physicians in epilepsy care during the COVID‐19 pandemic: a national‐level cross‐sectional survey in Japan. Epilepsy Behav. 2022;126:108487. 34922326 10.1016/j.yebeh.2021.108487 PMC9759923

[31] Ministry of Health Labour and Welfare . Time‐limited and special measures for medical treatment using telephones and information communication devices in the event of the spread of new coronavirus infections [in Japanese] (Administrative Notice, April 10, 2020). 2020. Available at: https://www.mhlw.go.jp/content/000620995.pdf

[32] Looi MK . Covid‐19: Japan declares state of emergency as Tokyo cases soar. BMJ. 2020;369:m1447. 32273382 10.1136/bmj.m1447

[33] Bhaskar S , Bradley S , Chattu VK , Adisesh A , Nurtazina A , Kyrykbayeva S , et al. Telemedicine across the Globe‐Position Paper from the COVID‐19 pandemic health system resilience PROGRAM (REPROGRAM) international consortium (Part 1). Front Public Health. 2020;16(8):556720. 10.3389/fpubh.2020.556720 PMC7596287 33178656

[34] Begley C , Basu R , Lairson D , Reynolds T , Dubinsky S , Newmark M , et al. Socioeconomic status, health care use, and outcomes: persistence of disparities over time. Epilepsia. 2011;52(5):957–64. 21320113 10.1111/j.1528-1167.2010.02968.x

[35] Blank LJ . Socioeconomic disparities in epilepsy care. Curr Opin Neurol. 2022;35(2):169–74. 35044956 10.1097/WCO.0000000000001031

[36] Bhaskar S , Nurtazina A , Mittoo S , Banach M , Weissert R . Editorial: telemedicine during and beyond COVID‐19. Front Public Health. 2021;16(9):662617. 10.3389/fpubh.2021.662617 PMC8007781 33796502

[37] Bhaskar S , Rastogi A , Menon KV , Kunheri B , Balakrishnan S , Howick J . Call for action to address equity and justice divide during COVID‐19. Front Psych. 2020;11:559905. 10.3389/fpsyt.2020.559905 PMC7744756 33343410

[38] Moura LM , Price M , Cole AJ , Hoch DB , Hsu J . Accuracy of claims‐based algorithms for epilepsy research: revealing the unseen performance of claims‐based studies. Epilepsia. 2017;58(4):683–91. 28199007 10.1111/epi.13691 PMC6592609

[39] Reid AY , St Germaine‐Smith C , Liu M , Sadiq S , Quan H , Wiebe S , et al. Development and validation of a case definition for epilepsy for use with administrative health data. Epilepsy Res. 2012;102(3):173–9. 22727659 10.1016/j.eplepsyres.2012.05.009

[40] Bullano MF , Kamat S , Willey VJ , Barlas S , Watson DJ , Brenneman SK . Agreement between administrative claims and the medical record in identifying patients with a diagnosis of hypertension. Med Care. 2006;44(5):486–90. 16641668 10.1097/01.mlr.0000207482.02503.55

