# Supplementary material for: Impact of COVID‐19 pandemic on epilepsy care in Japan: A national‐level multicenter retrospective cohort study
Source: Epilepsia Open. 2022 Jun 12;7(3):431–41. doi: 10.1002/epi4.12616 (PMC9348370; doi:10.1002/epi4.12616)
Supplement: Supplementary file 1 — Appendix S1 [file EPI4-7-431-s001.docx]

**Impact of COVID-19 pandemic on epilepsy care in Japan: A national-level multicenter retrospective cohort study**

Naoto Kuroda^1,2*^, Takafumi Kubota^1,3^, Toru Horinouchi^1,4^, Naoki Ikegaya^1,5*^, Yu Kitazawa^1,6^, Satoshi Kodama^1,7^, Izumi Kuramochi^1,8^, Teppei Matsubara^1,9^, Naoto Nagino^1,10^, Shuichiro Neshige^1,11^, Temma Soga^1,12^, Yutaro Takayama^1,13^, Daichi Sone^1,14^; IMPACT-J EPILEPSY (In-depth Multicenter analysis during Pandemic of Covid19 Throughout Japan for Epilepsy practice) study group (Kousuke Kanemoto^15^, Akio Ikeda^16^, Kiyohito Terada^17^, Hiroko Goji^15^, Shinji Ohara^18^, Koichi Hagiwara^19^, Takashi Kamada^19^, Koji Iida^20^, Nobutsune Ishikawa^21^, Hideaki Shiraishi^22^, Osato Iwata^23^, Hidenori Sugano^24^, Yasushi Iimura^24^, Takuichiro Higashi^25^, Hiroshi Hosoyama^25^, Ryosuke Hanaya^25^, Akihiro Shimotake^26^, Takayuki Kikuchi^27^, Takeshi Yoshida^28^, Hiroshi Shigeto^29^, Jun Yokoyama^30^, Takahiko Mukaino^30^, Masaaki Kato^31^, Masanori Sekimoto^31^, Masahiro Mizobuchi^32^, Yoko Aburakawa^32^, Masaki Iwasaki^13^, Eiji Nakagawa^33^, Tomohiro Iwata^34^, Kentaro Tokumoto^35^, Takuji Nishida^35^, Yukitoshi Takahashi^35^, Kenjiro Kikuchi^36^, Ryuki Matsuura^36^, Shin-ichiro Hamano^36^, Ayataka Fujimoto^37^, Hideo Enoki^37^, Kyoichi Tomoto^38^, Masako Watanabe^39^, Youji Takubo^39^, Toshihiko Fukuchi^40^, Hidetoshi Nakamoto^10^, Yuichi Kubota^10^, Naoto Kunii^41^, Yuichiro Shirota^42^, Eiichi Ishikawa^43^, Nobukazu Nakasato^12^, Taketoshi Maehara^44^, Motoki Inaji^44^, Shunsuke Takagi^45^, Takashi Enokizono^46^, Yosuke Masuda^47^, Takahiro Hayashi^5^)

**Method S1: Ethics committee approval in each facility.**

**Figure S1: The transition of new COVID-19 cases across Japan in 2020.**

**Figure S2: Participating facilities.**

**Figure S3: Box plots of monthly data values for each subgroup analysis.**

**Table S1: Linear mixed model analysis to characterize associated factors with each primary outcome.**

**Table S2: Linear mixed model analysis to characterize associated factors with each subgroup of the number of visits by outpatients with epilepsy.**

**Table S3: Linear mixed model analysis to characterize associated factors with each subgroup of the number of cases of telemedicine in epilepsy.**

**Method S1: Ethics committee approval in each facility.**

(Alphabetical order)

Aichi Medical University: Approved by the ethics committee in the facility.

Fukuoka Sanno Hospital: Approved by the ethics committee in the facility.

Hiroshima University: Approved by the ethics committee in the facility.

Hokkaido University: Approved by the ethics committee in the facility.

Iwata Clinic: Ethics approval was not required in the facility.

Juntendo University: Approved by the ethics committee in the facility.

Kagoshima University: Approved by the ethics committee in the facility.

Kyoto University: Approved by the ethics committee in the facility.

Kyushu University: Approved by the ethics committee in the facility.

Musashino Kokubunji Clinic: Ethics approval was not required in the facility.

Nakamura Memorial Hospital: Approved by the ethics committee in the facility.

National Center of Neurology and Psychiatry: Approved by the ethics committee in the facility.

National Defense Medical College: Approved by the ethics committee in the facility.

NHO. Shizuoka Institute of Epilepsy and Neurological Disorders: Approved by the ethics committee in the facility.

Saitama Children’s Medical Center: Approved by the ethics committee in the facility.

Seirei Hamamatsu General Hospital: Approved by the ethics committee in the facility.

Shinjuku Neuro Clinic: Ethics approval was not required in the facility.

Suzukake Clinic: Ethics approval was not required in the facility.

The University of Tokyo: Approved by the ethics committee in the facility.

The University of Tsukuba: Approved by the ethics committee in the facility.

TMG Asaka Medical Center: Approved by the ethics committee in the facility.

Tohoku University: Ethics approval was not required in the facility.

Tokyo Medical and Dental University: Approved by the ethics committee in the facility.

Yokohama City University: Approved by the ethics committee in the facility.

**Figure S1: The transition of new COVID-19 cases across Japan in 2020.**


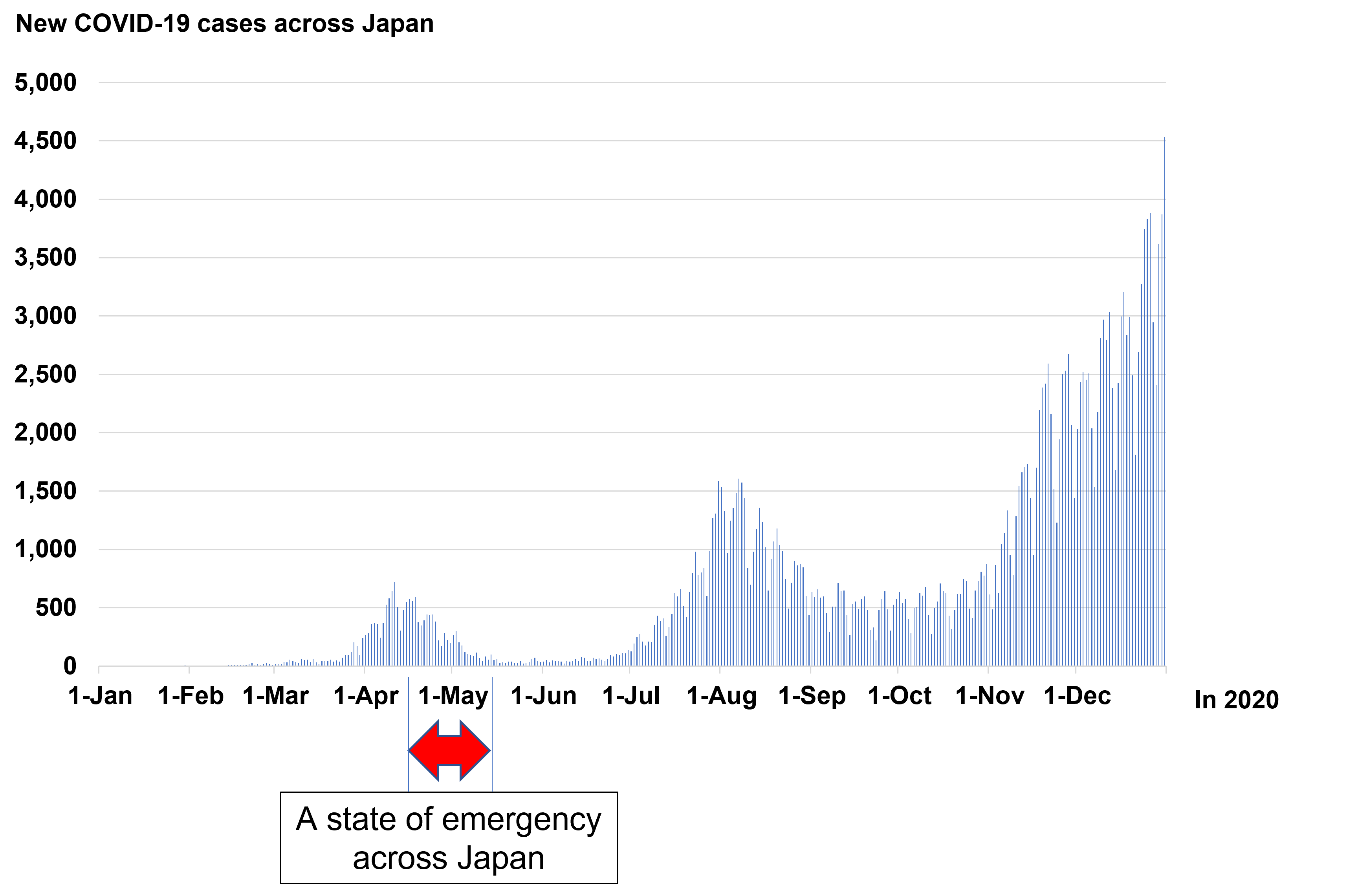


The bar graph shows the number of new COVID-19 cases in each day across Japan in 2020. Red arrow shows the period ordered a state of emergency across Japan.


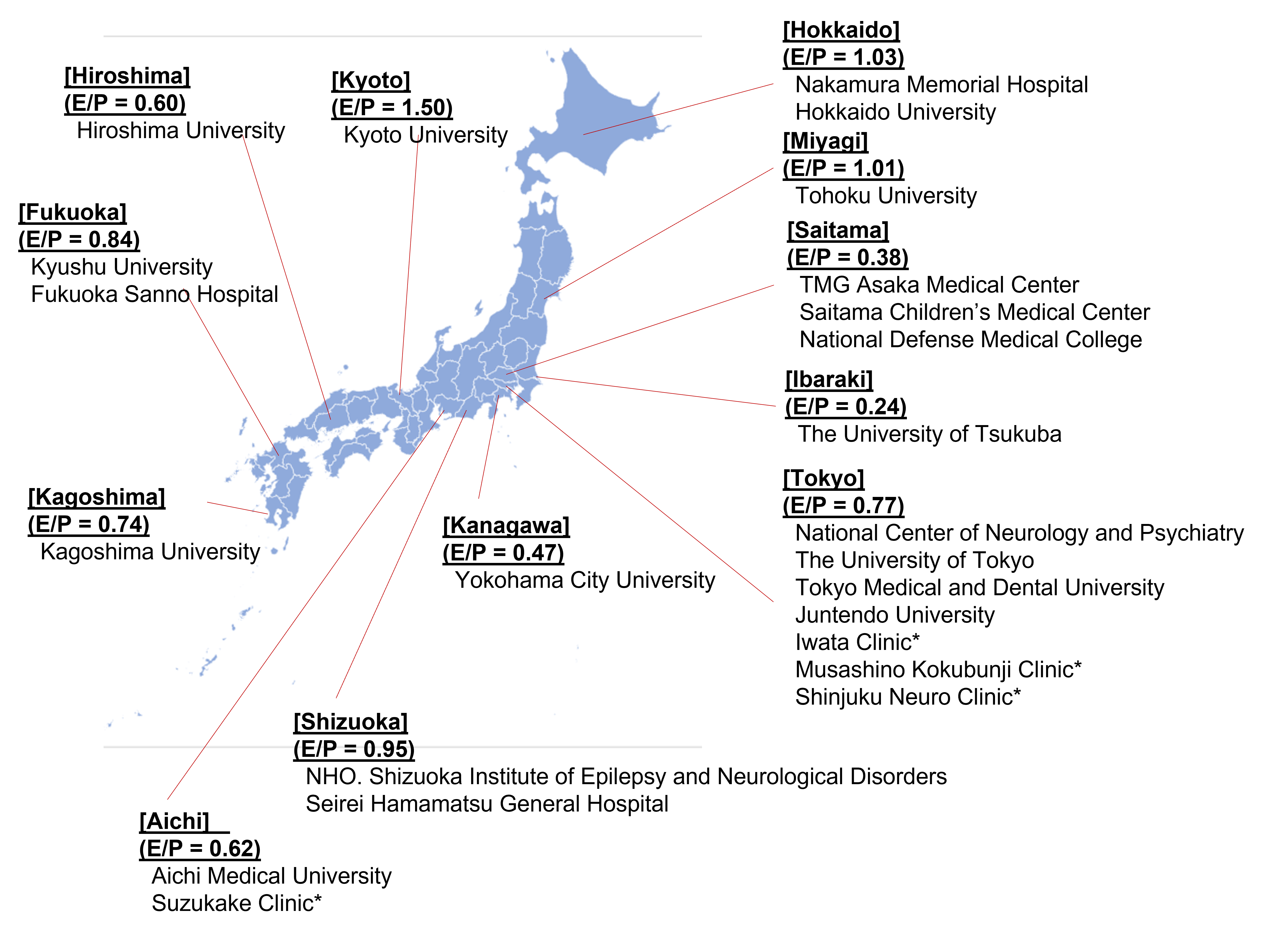
**Figure S2: Map of participating facilities.**

The map of Japan shows the prefectures of the participating facilities.

E/P: (Number of physicians with epilepsy board certificate) / (million population).

E/P across Japan was 0.62.

*: Clinic.


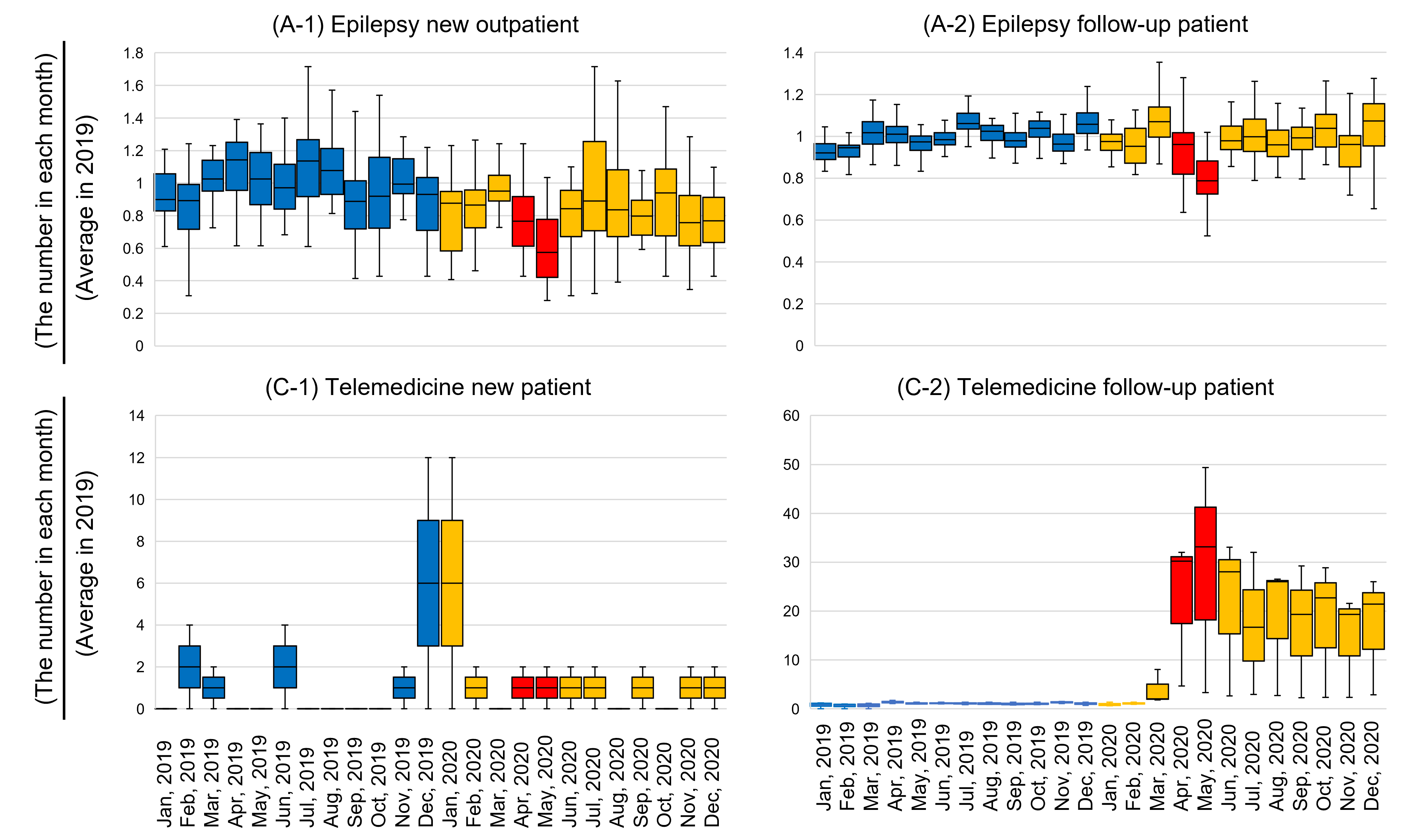
**Figure S3: Box plots of monthly data values for each subgroup analysis.**

The blue boxplots show the data for 2019. The orange boxplots show the data for 2020. The red boxplots show the data during the state of emergency (April–May 2020). (A-1) Normalized data of the monthly number of outpatient *new visits* with epilepsy by the 12-month average in 2019 in each facility. (A-2) Normalized data of the monthly number of outpatient *follow-up visits* with epilepsy by the 12-month average in 2019 in each facility. (C-1) Normalized data of the monthly number of *new visits* with telemedicine in epilepsy by the 12-month average in 2019 in each facility. (C-2) Normalized data of the monthly number of *follow-up visits* with telemedicine in epilepsy by the 12-month average in 2019 in each facility.

**Table S1: Linear mixed model analysis to characterize associated factors with each primary outcome.**

| Parameter | Coefficient | SE | df | t-value | P-value | 95% CI | |
| --- | --- | --- | --- | --- | --- | --- | --- |
|  |  |  |  |  |  | Lower Bound | Upper Bound |
| Epilepsy outpatient | | | | | | | |
| Facility  (0: Clinic, 1: Hospital) | -0.018 | 0.039 | 19.976 | -0.462 | 0.649 | -0.099 | 0.063 |
| Population | -9.20*10^-10^ | 3.17*10^-9^ | 20.932 | -0.290 | 0.775 | -7.52*10^-9^ | 5.68*10^-9^ |
| Number of board-certified physicians | 0.0002 | 0.002 | 19.980 | 0.092 | 0.927 | -0.005 | 0.005 |
| Year (0: 2019, 1: 2020) | -0.003 | 0.012 | 549.334 | -0.287 | 0.774 | -0.026 | 0.020 |
| Number of COVID19 cases in a previous month | -1.18*10^-6^ | 3.32*10^-6^ | 551.064 | -0.356 | 0.722 | -7.70*10^-6^ | 5.34*10^-6^ |
| State of emergency | -0.119 | 0.019 | 549.013 | -6.127 | **<0.001** | -0.157 | -0.081 |
| (Intercept) | 1.021 | 0.050 | 20.622 | 20.524 | **<0.001** | 0.917 | 1.124 |
| EEG outpatient | | | | | | | |
| Facility  (0: Clinic, 1: Hospital) | -0.013 | 0.046 | 20.007 | -0.290 | 0.775 | -0.109 | 0.082 |
| Population | -3.00*10^-9^ | 3.80*10^-9^ | 22.429 | -0.790 | 0.438 | -1.09*10^-8^ | 4.87*10^-9^ |
| Number of board-certified physicians | -0.0003 | 0.003 | 20.018 | -0.120 | 0.906 | -0.006 | 0.006 |
| Year (0: 2019, 1: 2020) | -0.107 | 0.022 | 549.880 | -4.912 | **<0.001** | -0.150 | -0.064 |
| Number of COVID19 cases in a previous month | -9.22*10^-6^ | 6.17*10^-6^ | 554.005 | -1.493 | 0.136 | -2.13*10^-5^ | 2.91*10^-6^ |
| State of emergency | -0.323 | 0.036 | 549.085 | -8.908 | **<0.001** | -0.394 | -0.252 |
| (Intercept) | 1.036 | 0.059 | 21.642 | 17.477 | **<0.001** | 0.913 | 1.159 |
| Telemedicine | | | | | | | |
| Facility  (0: Clinic, 1: Hospital) | 296.46 | 62.43 | 137 | 4.748 | **<0.001** | 173.00 | 419.92 |
| Population | 2.22*10^-5^ | 5.82*10^-6^ | 137 | 3.818 | **<0.001** | 1.07*10^-5^ | 3.37*10^-5^ |
| Number of board-certified physicians | -2.628 | 0.931 | 137 | -2.821 | **0.005** | -4.470 | -0.786 |
| Year (0: 2019, 1: 2020) | 26.08 | 11.947 | 137 | 2.183 | **0.031** | 2.455 | 49.703 |
| Number of COVID19 cases in a previous month | 0.007 | 0.004 | 137 | 1.697 | 0.092 | -0.001 | 0.016 |
| State of emergency | 129.15 | 20.61 | 137 | 6.267 | **<0.001** | 88.40 | 169.90 |
| (Intercept) | -333.38 | 82.42 | 137 | -4.045 | **<0.001** | -496.36 | -170.40 |
| Epilepsy inpatient | | | | | | | |
| Population | -3.68*10^-9^ | 4.93*10^-9^ | 19.905 | -0.746 | 0.464 | -1.40*10^-8^ | 6.61*10^-9^ |
| Number of board-certified physicians | -4.40*10^-5^ | 0.004 | 17.011 | -0.012 | 0.990 | -0.008 | 0.007 |
| Year (0: 2019, 1: 2020) | -0.041 | 0.029 | 457.876 | -1.405 | 0.161 | -0.098 | 0.016 |
| Number of COVID19 cases in a previous month | -3.75*10^-5^ | 9.69*10^-6^ | 462.708 | -3.866 | **<0.001** | -5.65*10^-5^ | -1.84*10^-5^ |
| State of emergency | -0.353 | 0.049 | 457.090 | -7.180 | **<0.001** | -0.450 | -0.257 |
| (Intercept) | 1.025 | 0.049 | 21.430 | 21.068 | **<0.001** | 0.924 | 1.126 |
| EEG monitoring | | | | | | | |
| Population | -1.52*10^-8^ | 8.86*10^-9^ | 17.487 | -1.713 | 0.104 | -3.38*10^-8^ | 3.48*10^-9^ |
| Number of board-certified physicians | -0.003 | 0.007 | 15.993 | -0.452 | 0.658 | -0.017 | 0.011 |
| Year (0: 2019, 1: 2020) | 0.013 | 0.040 | 434.409 | 0.326 | 0.744 | -0.066 | 0.093 |
| Number of COVID19 cases in a previous month | -3.81*10^-5^ | 1.32*10^-5^ | 436.832 | -2.888 | **0.004** | -6.39*10^-5^ | -1.22*10^-5^ |
| State of emergency | -0.247 | 0.068 | 434.034 | -3.627 | **<0.001** | -0.382 | -0.113 |
| (Intercept) | 1.121 | 0.088 | 18.204 | 12.727 | **<0.001** | 0.937 | 1.306 |
| Epilepsy surgery | | | | | | | |
| Population | -7.36*10^-9^ | 9.23*10^-9^ | 16.225 | -0.797 | 0.437 | -2.69*10^-8^ | 1.22*10^-8^ |
| Number of board-certified physicians | -0.003 | 0.007 | 11.995 | -0.508 | 0.620 | -0.018 | 0.011 |
| Year (0: 2019, 1: 2020) | 0.115 | 0.080 | 342.837 | 1.432 | 0.153 | -0.043 | 0.273 |
| Number of COVID19 cases in a previous month | -2.30*10^-5^ | 2.44*10^-5^ | 347.330 | -0.939 | 0.348 | -7.10*10^-5^ | 2.51*10^-5^ |
| State of emergency | -0.503 | 0.136 | 342.071 | -3.714 | **<0.001** | -0.770 | -0.237 |
| (Intercept) | 1.073 | 0.098 | 18.403 | 10.926 | **<0.001** | 0.867 | 1.279 |

SE: standard error

df: degree of freedom

CI: confidence interval

**Table S2: Linear mixed model analysis to characterize associated factors with each subgroup of the number of visits by outpatients with epilepsy.**

| Parameter | Coefficient | SE | df | t-value | P-value | 95% CI | |
| --- | --- | --- | --- | --- | --- | --- | --- |
|  |  |  |  |  |  | Lower Bound | Upper Bound |
| Epilepsy new outpatient | | | | | | | |
| Facility  (0: Clinic, 1: Hospital) | 0.039 | 0.115 | 20.002 | 0.339 | 0.74 | -0.201 | 0.280 |
| Population | -7.62*10^-9^ | 9.43*10^-9^ | 20.999 | -0.808 | 0.43 | -2.72*10^-8^ | 1.20*10^-8^ |
| Number of board-certified physicians | -0.009 | 0.007 | 20.006 | -1.294 | 0.21 | -0.025 | 0.006 |
| Year (0: 2019, 1: 2020) | -0.037 | 0.035 | 549.375 | -1.043 | 0.30 | -0.106 | 0.033 |
| Number of COVID19 cases in a previous month | -3.01*10^-5^ | 1.01*10^-5^ | 551.172 | -2.991 | **0.003** | -4.98*10^-5^ | -1.03*10^-5^ |
| State of emergency | -0.174 | 0.059 | 549.040 | -2.960 | **0.003** | -0.290 | -0.059 |
| (Intercept) | 1.078 | 0.148 | 20.676 | 7.298 | **<0.001** | 0.770 | 1.385 |
| Epilepsy follow-up outpatient | | | | | | | |
| Facility  (0: Clinic, 1: Hospital) | -0.016 | 0.038 | 19.971 | -0.423 | 0.68 | -0.096 | 0.063 |
| Population | -4.84*10^-10^ | 3.11*10^-9^ | 20.993 | -0.155 | 0.88 | -6.96*10^-9^ | 5.99*10^-9^ |
| Number of board-certified physicians | 0.001 | 0.002 | 19.975 | 0.268 | 0.79 | -0.004 | 0.006 |
| Year (0: 2019, 1: 2020) | 0.001 | 0.012 | 549.354 | 0.098 | 0.92 | -0.022 | 0.024 |
| Number of COVID19 cases in a previous month | 5.81*10^-7^ | 3.36*10^-6^ | 551.200 | 0.173 | 0.86 | -6.02*10^-6^ | 7.18*10^-6^ |
| State of emergency | -0.120 | 0.020 | 549.010 | -6.079 | **<0.001** | -0.158 | -0.081 |
| (Intercept) | 1.014 | 0.049 | 20.662 | 20.790 | **<0.001** | 0.912 | 1.115 |

SE: standard error

df: degree of freedom

CI: confidence interval

**Table S3: Linear mixed model analysis to characterize associated factors with each subgroup of the number of cases of telemedicine in epilepsy.**

| Parameter | Coefficient | SE | df | t-value | P-value | 95% CI | |
| --- | --- | --- | --- | --- | --- | --- | --- |
|  |  |  |  |  |  | Lower Bound | Upper Bound |
| New patient with telemedicine | | | | | | | |
| Facility  (0: Clinic, 1: Hospital) | - | - | - | - | - | - | - |
| Population | -1.09*10^-7^ | 2.09*10^-6^ | 43 | -0.052 | 0.959 | -4.31*10^-6^ | 4.10*10^-6^ |
| Number of board-certified physicians | - | - | - | - | - | - | - |
| Year (0: 2019, 1: 2020) | 0.373 | 0.869 | 43 | 0.429 | 0.670 | -1.379 | 2.125 |
| Number of COVID19 cases in a previous month | -0.001 | 0.003 | 43 | -0.515 | 0.610 | -0.006 | 0.004 |
| State of emergency | -0.325 | 1.469 | 43 | -0.221 | 0.826 | -3.289 | 2.638 |
| (Intercept) | 1.322 | 6.329 | 43 | 0.209 | 0.836 | -11.44 | 14.09 |
| Follow-up patient with telemedicine | | | | | | | |
| Facility  (0: Clinic, 1: Hospital) | 237.73 | 232.37 | 89 | 1.023 | 0.309 | -223.976 | 699.438 |
| Population | 1.41*10^-5^ | 2.68*10^-5^ | 89 | 0.526 | 0.600 | -3.91*10^-5^ | 6.73*10^-5^ |
| Number of board-certified physicians | -4.053 | 5.481 | 89 | -0.740 | 0.461 | -14.944 | 6.837 |
| Year (0: 2019, 1: 2020) | 29.07 | 12.445 | 89 | 2.336 | **0.022** | 4.343 | 53.801 |
| Number of COVID19 cases in a previous month | 0.007 | 0.004 | 89 | 1.931 | 0.057 | -2.09*10^-4^ | 0.015 |
| State of emergency | 74.034 | 21.075 | 89 | 3.513 | **<0.001** | 32.159 | 115.909 |
| (Intercept) | -214.923 | 379.759 | 89 | -0.566 | 0.573 | -969.495 | 539.649 |

SE: standard error

df: degree of freedom

CI: confidence interval
